# Massive Pressure Amplification by Stimulated Contraction of Mesoporous Frameworks**

**DOI:** 10.1002/anie.202100549

**Published:** 2021-05-03

**Authors:** Volodymyr Bon, Simon Krause, Irena Senkovska, Nico Grimm, Dirk Wallacher, Daniel M. Többens, Stefan Kaskel

**Affiliations:** ^1^ Department of Inorganic Chemistry I Technische Universität Dresden Bergstrasse 66 01069 Dresden Germany; ^2^ Centre for Systems Chemistry Stratingh Institute for Chemistry University of Groningen Nijenborgh 4 9747 AG Groningen The Netherlands; ^3^ Helmholtz-Zentrum Berlin für Materialien und Energie Hahn-Meitner-Platz 1 14109 Berlin Germany

**Keywords:** DUT-49, metal–organic frameworks, negative gas adsorption, pneumatics, pressure amplification

## Abstract

Herein we demonstrate mesoporous frameworks interacting with carbon dioxide leading to stimulated structural contractions and massive out‐of‐equilibrium pressure amplification well beyond ambient pressure. Carbon dioxide, a non‐toxic and non‐flammable working medium, is promising for the development of pressure‐amplifying frameworks for pneumatic technologies and safety systems. The strong interaction of the fluid with the framework even contracts DUT‐46, a framework hitherto considered as non‐flexible. Synchrotron‐based in situ PXRD/adsorption experiments reveal the characteristic contraction pressure for DUT‐49 pressure amplification in the range of 350–680 kPa. The stimulated framework contraction expels 1.1 to 2.4 mmol g^−1^ CO_2_ leading to autonomous pressure amplification in a pneumatic demonstrator system up to 428 kPa. According to system level estimations even higher theoretical pressure amplification may be achieved between 535 and 1011 kPa.

## Graphical abstract

Negative Gas Adsorption is a unique counterintuitive phenomenon of responsive metal–organic frameworks hitherto only observed below atmospheric pressure. Carbon dioxide stimulates contraction at high absolute pressure inducing massive pressure amplification exceeding 500 kPa.

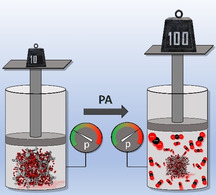

The principle of Le Châtelier and Braun elaborated between 1884 and 1888 is famous and among the most important guiding principles in chemistry.^[1]^ A disturbance in a system will cause a shift to counterbalance the effect of this disturbance. Pressurizing a gas phase in contact with a porous solid will always result in a spontaneous pressure drop and the system responds by adsorbing the fluid inside the pores to counterbalance the outer gas pressure increase. The discovery of spontaneous gas desorption stimulated by pressure increase does not follow this principle, instead it is a counterintuitive phenomenon far out of equilibrium.^[2]^ However, the initially observed gas release initiated only a rather tiny pressure amplification of a few kPa and the operating conditions (methane, 111 K) were considered as unpractical so far. In recent years the understanding of counterintuitive pressure amplification (PA) by porous frameworks (also termed “Negative Gas Adsorption”, NGA) has been significantly advanced. Only mesoporous switchable frameworks with a pore size above 2 nm lead to PA.^[3]^ The micromechanics require stiff building blocks which only buckle upon exertion of a critical adsorption stress.^[4]^ A wide range of fluids can stimulate PA and the operating temperature is closely connected to the critical temperature of the fluid indicating fluid nucleation to play an important role in controlling the out‐of‐equilibrium stability against perturbation.^[5]^

Switchable metal–organic frameworks (MOFs) were serendipitously discovered in 2001^[6]^ and today are at the forefront of porous materials research.^[7]^ They counterbalance a gas pressure increase by the inclusion of guest molecules adapting their pore size, which in some cases results in tremendous cell volume changes.[^7a^, ^7b^, ^7f^, ^8^] Beyond guest‐induced switchability,^[7c]^ also temperature,^[9]^ UV‐ and visible light,^[10]^ electric and magnetic fields^[11]^ have been demonstrated as external stimuli. The combination of flexibility, crystallinity, and porosity renders switchable MOFs an important class of materials for advanced applications such as gas storage,^[12]^ threshold sensors,^[13]^ or molecular separation.^[14]^ Mechanical aspects make bistable frameworks promising shock absorbers or nanosprings.^[15]^

However, only a very limited number of switchable MOFs demonstrate counterintuitive out‐of‐equilibrium pressure amplification phenomena. The prototypical pressure amplifying frameworks, DUT‐49(Cu) analogues, are based on porous cuboctahedral metal–organic polyhedral (MOP) cages (Figure 1 b), built of copper paddle‐wheels and 9*H*‐carbazole‐3,6‐dicarboxylates,^[16]^ assembled in a ccp‐analog packing (Figure 1 a) with mesoporous tetrahedral and octahedral cages^[17]^ (Figure 1 c,d) of adjustable dimensions defined by the spacer length between adjacent metal–organic polyhedra (Figure 1 e).^[3]^ During pore filling of the large mesopores the systems traverse through a metastable state, an overloaded pore, shortly before a massive contraction is initiated by the cohesive forces of the fluid resulting overall in the expulsion of gas moles Δ*n*_NGA_ from the pores (Negative Gas Adsorption) and the expelled gas molecules increase the total pressure of the closed system (pressure amplification).

**Figure 1:**
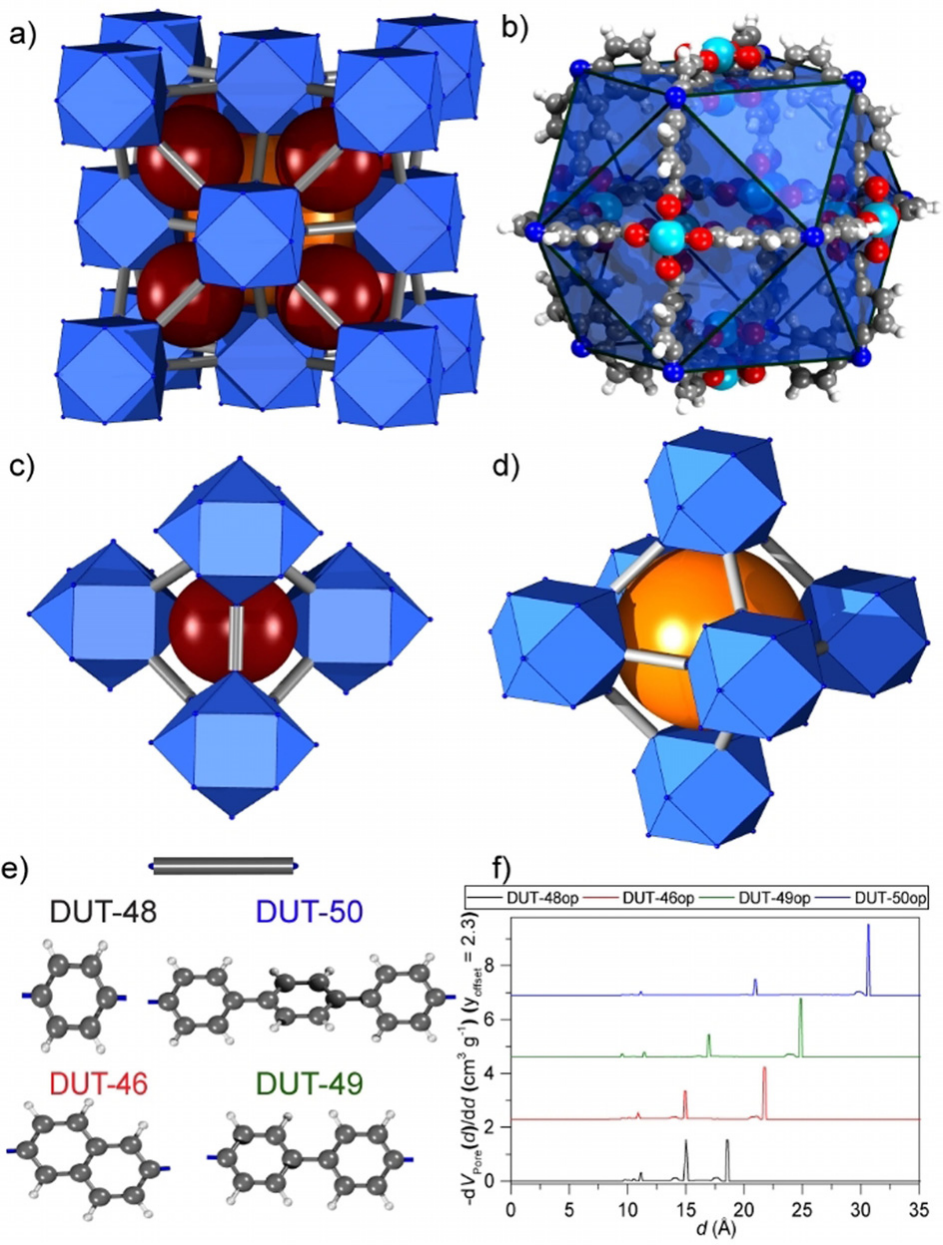
a–d) Pore structure of carbazole‐MOP‐based MOFs; e) spacers used in the ligands of DUT‐48, 46, 49, and 50; f) theoretical pore‐size distribution for open‐pore phases of DUT‐48, 46, 49, and 50.

The exotic operation conditions (p<30 kPa) of this counterintuitive phenomenon so far hampered an implementation in real‐world applications, typically requiring pressure amplification in the range of 0.1–1 MPa.^[5]^ In this sense there is a strong need for the identification of non‐toxic, non‐flammable fluids inducing PA above ambient atmospheric pressure. The latter would enable the exploitation of PA materials as a technology platform.

Herein, we demonstrate pressure amplification beyond 100 kPa for the first time using CO_2_ as a stimulus. Carbon dioxide is a non‐flammable, non‐toxic, and inexpensive gas, the molecule of which shows a quadrupolar moment and high adsorption enthalpy.^[18]^ It is an ideal fluid for the operation of pneumatic devices and machines such as dampers, fire extinguishers, or life jackets,^[19]^ and more recently is gaining attention in the field of pneumatic logic circuits for electronics‐free control of soft‐legged pneumatic robots.^[20]^ This motivated us to assess the value of carbon dioxide for high‐pressure amplification by NGA materials.

To achieve this goal, we studied pressure amplification by CO_2_ using a series of isoreticular frameworks: DUT‐48(Cu), DUT‐46(Cu), DUT‐49(Cu), and DUT‐50(Cu) in an industrially relevant temperature range of 195–298 K with the ambition to maximize the magnitude of PA. Advanced in situ X‐ray diffraction analysis provides important insights into the contraction mechanisms of the solids. Finally, we establish guidelines for the integration of PA materials into pneumatic systems.

The metal–organic frameworks DUT‐48(Cu)/PCN‐81,^[21]^ DUT‐46(Cu), DUT‐49(Cu), and DUT‐50(Cu) belong to an isoreticular series in which the spacer, linking the MOPs, varies in length (48: phenyl, 46: naphthyl, 49: biphenyl, 50: terphenyl, Figure 1 e).^[3]^ Phase purity of as‐made and supercritically desolvated samples was confirmed by powder X‐ray diffraction. SEM analysis reveals a particle size ranging from 2 to 14 μm (Supporting Information, Figure S1). Upon ligand elongation the pore size increases step‐wise from 1.5 to 2.1 nm for the tetrahedral voids and 1.8 to 3.1 nm for the octahedral voids (Figure 1 f).

A characteristic feature of all these structures is bistability with two minima in the free energy profiles, but the relative energy of the minima varies widely with the spacer length. The energetic difference (Δ*F*, Table 1) of the two minima is higher for the shorter ligands and they buckle only under high adsorption stress. The contraction of the open‐pore framework (*op*) thus requires significant energy and the magnitude is dominated by the buckling deformation of the ligand.^[3]^ Therefore, weakly interacting fluids are not able to stimulate structural contraction in these solids. In case of nitrogen physisorption at 77 K hysteretic isotherms and NGA were only observed for DUT‐49 and DUT‐50 (Figure S4a, Supporting Information). In contrast, DUT‐48 and DUT‐46 show reversible type Ib isotherms, typical for rigid porous solids with small mesopores. The difference in adsorption behavior is a result of the higher mechanical stiffness of DUT‐46 and DUT‐48 which do not contract under the marginal adsorption‐induced stress generated by N_2_ adsorption at 77 K.

**Table 1:** Experimental and theoretical mechanical properties of investigated MOFs.^[3]^

MOF	Geometric pore volume *op*/*cp* phase [cm^3^ g^−1^]	Unit cell volume *op* phase *V* [nm^3^]	Unit cell volume *cp* phase *V* [nm^3^]	*ΔF* guest‐free *op* and *cp* frameworks [kJ mol^−1^]
DUT‐48	1.67/0.39	63.044	35.287	892
DUT‐46	1.89/0.46	78.402	41.421	793
DUT‐49	2.64/0.54	96.071	45.882	768
DUT‐50	3.45/0.74	142.645	58.185	598

For achieving high‐pressure amplification we applied CO_2_ as a probe molecule in physisorption experiments at 195 K. In the phase diagram of CO_2_, the triple point is located at 216.5 K and 0.51 MPa. However, according to theoretical and experimental studies adsorbed CO_2_ at 195 K behaves as an undercooled fluid, showing strong fluid–fluid interactions and in the temperature range of 210–240 K the condensation of CO_2_ in mesopores was observed.^[22]^

In order to analyze the impact of adsorption stress exerted by CO_2_ on the series of DUT‐49‐type frameworks, we first systematically studied the isoreticular series of DUT‐48, ‐46, ‐49, and ‐50 at 195 K (Figure S4b, Supporting Information). Although the free energy profiles of these isoreticular frameworks show a local minimum with reduced cell volume, hitherto guest‐induced switchability was only observed for DUT‐49 and DUT‐50. In contrast, DUT‐48 and DUT‐46, containing quite stiff linkers appeared as rigid and showed no contraction in the presence of other probe molecules at all temperatures tested so far.

DUT‐48 in the presence of CO_2_ at 195 K shows no indications of network switchability, the isotherm shows a distinct step at *p*/*p*_0_=0.25 (which is most likely not associated with the flexibility)^[22b]^ reaching a plateau at *p*/*p*_0_=0.3 with saturation capacity of 48.8 mmol g^−1^ (Figure S4b, Supporting Information). In order to prove whether this step is caused by structural deformation, in parallel to the adsorption experiment we collected PXRD patterns at 32 pressure points using a recently developed laboratory instrumentation. Interestingly, we detect a narrow H1‐type hysteresis, however no structural deformation is observed (Figure S3, Supporting Information). The pore volume calculated at *p*/*p*_0_=0.8 reaches 1.906 cm^3^ g^−1^, which matches the value calculated from the nitrogen isotherm at 77 K.

Surprisingly, the CO_2_ physisorption isotherm of DUT‐46 shows relatively low uptake in saturation after a step at *p*/*p*_0_=0.4 (Figure S4b, Supporting Information), reaching 31.5 mmol g^−1^ (*V*_p_=1.18 cm^3^g^−1^ at *p*/*p*_0_=0.8), which is unexpected compared with the porosity calculated from the crystal structure (*V_p_*=1.89 cm^3^ g^−1^, Table 1)^[3]^ and nitrogen physisorption (*V_p_*=2.132 cm^3^g^−1^ at *p*/*p*_0_=0.8). In addition, the desorption branch of the isotherm does not follow the adsorption branch at *p*/*p*_0_ below 0.4 (Figure S4b, Supporting Information), indicating adsorption‐induced changes of the pore system and structural transformations of the framework. Similar features and hystereses are observed for DUT‐49 and DUT‐50 with characteristic steps at *p*/*p*_0_=0.5 and *p*/*p*_0_=0.7 and small saturation uptakes of only 20.6 and 22.3 mmol g^−1^ at *p*/*p*_0_=0.8 (corresponding to a specific pore volume of only *V*_*p(DUT‐49)*_=0.76 cm^3^ g^−1^, *V*_*p(DUT‐50)*_=0.82 cm^3^ g^−1^) in contrast to porosity estimated from nitrogen adsorption isotherms (*V*_*p(DUT‐49)*_=2.82 cm^3^ g^−1^, *V*_*p(DUT‐50)*_=3.67 cm^3^ g^−1^; Figure S4b, Table S1, Supporting Information).

To investigate potential framework transitions in the adsorption process, the physisorption of CO_2_ on DUT‐46 and DUT‐49 was analyzed by advanced in situ X‐ray powder diffraction at KMC‐2 beamline of the BESSY‐II synchrotron in parallel to the adsorption and desorption of CO_2_ at 195 K (Figure 2).^[7d]^

**Figure 2:**
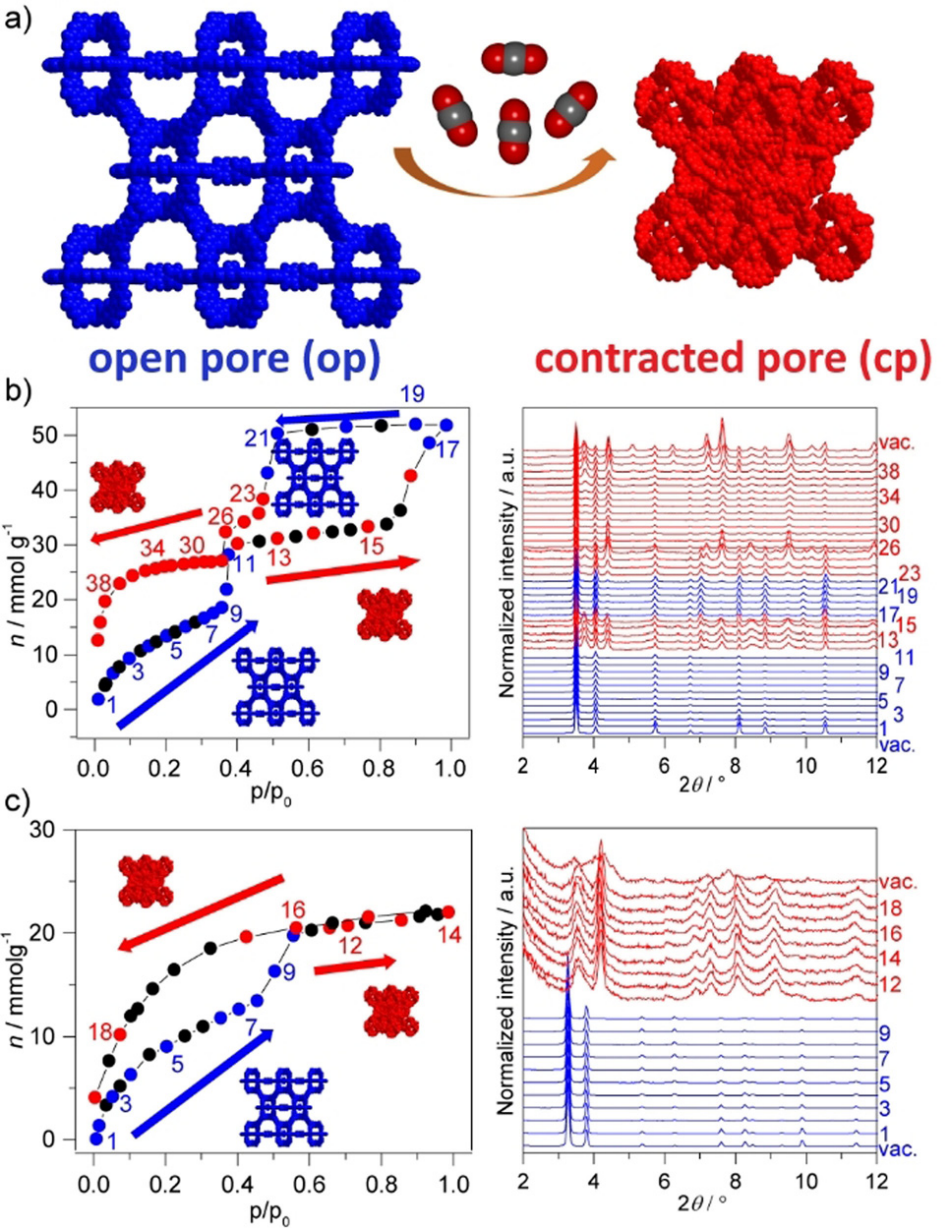
a) Structural contraction from open‐pore (blue) to contracted‐pore (red) phase, followed by in situ PXRD during CO_2_ adsorption at 195 K on b) DUT‐46 and c) DUT‐49 (numbers and color codes in the isotherm and PXRD patterns indicate the parallel measurements).

In situ studies clearly reveal pronounced structural contractions for both frameworks at a distinct relative pressures (*p/p_0_*=0.45 for DUT‐46 and 0.50 for DUT‐49) (Figure 2 a). In case of DUT‐46, the isotherm measured in situ shows an additional step at *p*/*p*_0_=0.9 and a second hysteresis, absent in the ex situ experiment. This second step can be assigned to the reopening of the structure (Figure 2 b) during adsorption at increasing relative pressure. Powder X‐ray diffraction (PXRD) patterns measured upon adsorption before reaching the plateau at *p*/*p*_0_=0.4 (PXRDs 1–11) indicated the *op* phase of the DUT‐46. Along the plateau (0.4<*p*/*p*_0_<0.9 in adsorption), a set of peaks different from the *op* phase appears which can be assigned to the formation of a contracted *cp* state (PXRDs 12–16), indicating the presence of phase mixture in this pressure range. Peaks assigned to the *cp* phase disappeared at the second step and only the *op* phase is detected at saturation at *p*/*p*_0_=0.98, which indicates reversible reopening (PXRDs 17–22). Again, *cp* and *op* phase mixtures appear along the desorption branch at *p*/*p*_0_≤0.45 (PXRDs 23–40). This finding is remarkable as it demonstrates for the first time adsorption‐induced switchability of DUT‐46, a network hitherto considered as non‐flexible due to the high mechanical stiffness. No other molecular stimulus investigated so far was able to induce structural contraction as type I isotherms were observed for N_2_ (77 K), Ar (87 K), CH_4_ (111 K), and *n*‐butane (273–298 K) characteristic for non‐responsive frameworks.^[3]^ For DUT‐49 a similar adsorption‐induced deformation mechanism is recorded as discussed for DUT‐46. Firstly, at a relative pressure of 0.55 (PXRDs 11–14), the framework contracts into the pure *cp* phase (Figure 2 c). However, in contrast to DUT‐46, no reopening is observed for DUT‐49 even at *p*/*p*_0_>0.98 and the *cp* phase is retained throughout the entire desorption process (PXRDs 15–19). This structural evolution explains the reduced adsorption capacity for DUT‐49 at elevated relative pressure. Interestingly, the structures of CO_2_‐loaded *cp* phases of DUT‐46 and DUT‐49 show an additional reflection at *2θ* between 3.5 and 3.6° (Figure 2 c), which was not observed in earlier studies using methane as a stimulus at 111 K. Detailed analysis of PXRD patterns of CO_2_‐filled *cp* phases indicates a lower symmetry (*P*2_1_3) to be the origin. Unfortunately, detailed structural refinement of *cp* phases is challenging because of *op*/*cp* phase mixture in case of DUT‐46 and broadening of the reflections in case of DUT‐49. However, comparison of experimentally measured PXRD patterns with corresponding simulated PXRDs for CO_2_‐loaded DUT‐46*cp* and DUT‐49*cp* structures indicates a good agreement in terms of positions and intensities (Figure S2, Supporting Information). Specific details of the crystal structures of the *cp* phases are discussed in Supporting Information section 2. A possible reason explaining the adsorption characteristics of CO_2_ at 195 K could be the strong intermolecular interactions between the CO_2_ molecules in the pores of the *cp* phase after contraction and enhanced host–guest interactions that hinder reopening of the structure.

The findings from adsorption and in situ PXRD experiments at 195 K motivated us to expand physisorption experiments on DUT‐46, DUT‐49, and DUT‐50 to a wider temperature range close and beyond the triple point of CO_2_ (Figure S5, Supporting Information). Adsorption of CO_2_ on DUT‐46 at 210, 220, and 230 K shows a gradual shift of the mesopore filling step from *p*/*p*_0_=0.4 towards lower relative pressures of *p*/*p*_0_=0.3 (210 K) and *p*/*p*_0_=0.25 (220 and 230 K). This effect confirms the changes in the guest–guest interactions between CO_2_ molecules in the pores, which are obviously attributed to the change of the adsorbed state of the fluid beyond the triple point.^[22b]^ All isotherms show a saturation uptake, expected for the pore volume derived from nitrogen physisorption at 77 K (Figure S5a, Table S1, Supporting Information). The isotherms at 220 and 230 K are completely reversible and show no indications of framework structural phase transitions. In the case of DUT‐49, isotherms measured in the temperature range between 220 and 250 K show similar behavior in terms of the shift of the adsorption step towards the lower *p*/*p*_0_ values (Figure S5b, Supporting Information). However, the isotherms measured in the range of 220–240 K show hystereses and isotherms typical for DUT‐49 PA transitions.^[2]^ A characteristic plateau in the relative pressure range of 0.3–0.7 indicates the contraction to a *cp* phase. After reopening, the solid reaches again an adsorption capacity of 55–70 mmol g^−1^. DUT‐50 follows the same trend as observed for DUT‐49 (Figure S5c, Supporting Information), however, the transition pressure is shifted towards higher values as it may be expected for larger pores.^[3]^

These observations motivated us to apply DUT‐49 and DUT‐50 as suitable pressure‐amplifying solids using CO_2_ at 230–240 K (Figure 3). In a custom‐built pressure amplifier (for details see section 2.5, Figure S13a, Supporting Information), we demonstrate the validity of the theoretically estimated pressure amplification values. After reaching *p_NGA_*=340 kPa, the pressure in the adsorption cell spontaneously increased reaching 428 kPa within 360 s (Figure 3 d). Such massive pressure amplification up to 428 kPa has never been reported before, demonstrating the effectiveness of pressure‐amplifying materials at pressure levels well beyond ambient pressure relevant for pneumatic systems (Figure S13, Supporting Information). Moreover, repeatable pressure amplification was demonstrated in a second cycle after reopening the framework. In the second cycle *p_NGA_* is only slightly shifted to 354 kPa and the overall pressure reaches 425 kPa in 470 s (Figure S13c, Supporting Information), an almost identical performance compared to the first run. A full CO_2_ adsorption isotherm at 230 K was recorded after reopening reaching *Δn_NGA_*=1.3 mmolg^−1^ and full capacity in terms of the pore volume compared to the first run (Figure S13d, Supporting Information). These results show for the first time dynamic framework materials capable of generating high pressures well beyond 100 kPa.

**Figure 3:**
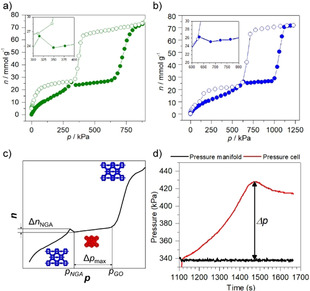
Adsorption isotherms of carbon dioxide on DUT‐49 at a) 230 K, b) DUT‐50 at 240 K; c) pressure amplification phenomenon during isothermal adsorption; d) pressure amplification in the adsorption demonstrator experiment at 230 K.

In summary, we identified conditions for massive pressure amplification by porous frameworks based on an isoreticular platform of PA frameworks, namely DUT‐48, DUT‐46, DUT‐49, and DUT‐50, offering a gradual increase in the pore size and specific pore volume. The mechanism behind this, an adsorption‐stimulated contraction, was analyzed by in situ X‐ray diffraction in the presence of CO_2_ at 195 K. The adsorption stress exerted by CO_2_ even contracts the relatively stiff DUT‐46, a framework hitherto considered as non‐deformable in the presence of other adsorbate molecules such as N_2_, CH_4_, and *n*‐butane. The strong interaction of CO_2_ with the host framework also leads to contraction of DUT‐49 and DUT‐50, however, reopening and breathing is suppressed in these less rigid systems. Breathing is only observed at higher temperatures above the carbon dioxide triple point. The gas release reaches 1.1–2.4 mmol g^−1^ at 230–240 K, leading to high absolute pressures for the PA transitions. The maximal estimated pressure operation window ranges from 350 to 600 kPa for DUT‐49 at 230 K, and from 538 and 680 kPa to 1000 kPa for DUT‐49 and DUT‐50 at 240 K, respectively. These estimations are fully validated by experimental demonstration of pressure amplification up to 428 kPa. Our observations provide fundamental insights for the understanding of porous systems far from equilibrium and represent a decisive step towards developing pressure amplifying frameworks for the integration in pneumatic architectures and safety systems.^[23]^

## Conflict of interest

The authors declare no conflict of interest.

## Supporting information

As a service to our authors and readers, this journal provides supporting information supplied by the authors. Such materials are peer reviewed and may be re‐organized for online delivery, but are not copy‐edited or typeset. Technical support issues arising from supporting information (other than missing files) should be addressed to the authors.

Supplementary
