# Supplementary material for: Massive Pressure Amplification by Stimulated Contraction of Mesoporous Frameworks
Source: Angew Chem Int Ed Engl. 2021 May 3;60(21):11735–9. doi: 10.1002/anie.202100549 (PMC8251781; doi:10.1002/anie.202100549)
Supplement: Supplementary file 1 — Supplementary [file ANIE-60-11735-s001.pdf]

Angewandte Chemie
Eine Zeitschrift der Gesellschaft Deutscher Chemiker

Supporting Information

Massive Pressure Amplification by Stimulated Contraction of Mesoporous Frameworks**

Volodymyr Bon, Simon Krause, Irena Senkovska, Nico Grimm, Dirk Wallacher, Daniel M. Többens, and Stefan Kaskel**

anie_202100549_sm_miscellaneous_information.pdf

Supporting Information

©Wiley-VCH 2019
69451 Weinheim, Germany

Abstract: Herein we demonstrate mesoporous frameworks interacting with carbon dioxide leading to stimulated structural contractions and massive out-of-equilibrium pressure amplification well beyond ambient pressure. Carbon dioxide, a non-toxic and non-flammable working medium is promising for the development of pressure amplifying frameworks for pneumatic technologies and safety systems. The strong interaction of the fluid with the framework even contracts DUT-46 (DUT = Dresden University of Technology), a framework hitherto considered as non-flexible. Synchrotron-based in situ PXRD/adsorption experiments reveal the characteristic contraction pressure for DUT-49 pressure amplification in the range of 350 - 680 kPa. The stimulated framework contraction expels 1.1 to 2.4 mmol g^{-1} CO_2 leading to autonomous pressure amplification in a pneumatic demonstrator system up to 428 kPa. According to system level estimations even higher theoretical pressure amplification may be achieved between 535 kPa and 1011 kPa.

DOI: 10.1002/anie.2016XXXXX

Table of Contents

1. Experimental procedures
    1.1. MOF synthesis
    1.2. Physical measurements
    1.3. In situ PXRD in parallel to physisorption of CO_2

2. Results and Discussions
    2.1. Analysis of *in situ* PXRD patterns measured upon adsorption of CO_2
    2.2. Physisorption of N_2 (77K) and CO_2 on DUT-46, DUT-49 and DUT-50
    2.3. Analysis of adsorption profile for NGA
    2.4. Calculation of the pressure amplification for DUT-49 and DUT-50
    2.5. Design of the pressure amplification experiment

1. Experimental Procedures

1.1. *MOF synthesis:*

Synthesis and characterization of ligands and MOFs used in the manuscript is described in our recent study.[1] SEM images and PXRD patterns, measured on DUT-48, DUT-46, DUT-49 and DUT-50 are given in figure S1.

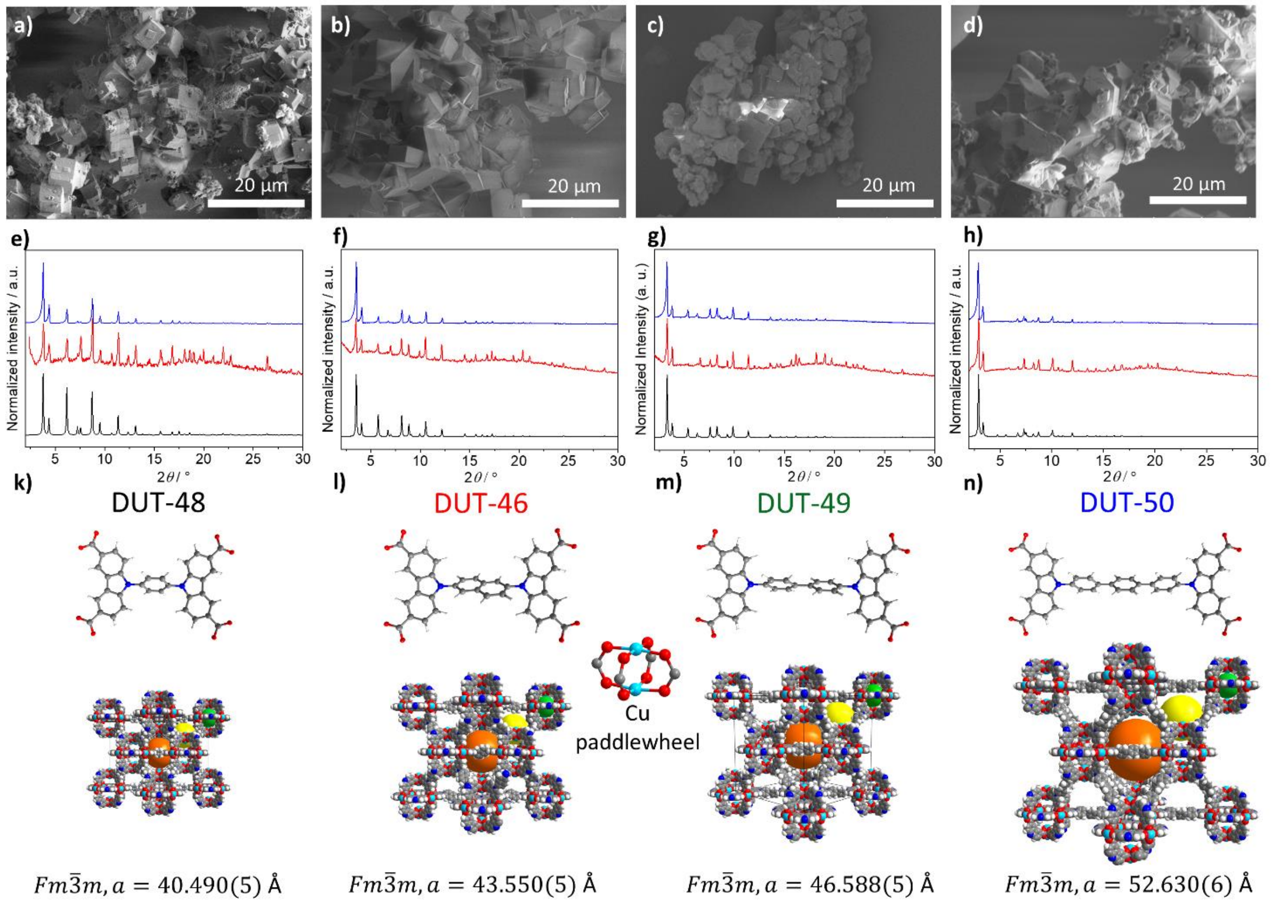

Figure S1. SEM images of DUT-48 (a), DUT-46 (b), DUT-49 (c), DUT-50); PXRD patterns measured on DUT-48 (e), DUT-46 (f), DUT-49 (g) and DUT-50 (h) (colour codes: calculated - black, as made – red, desolvated – blue lines); building blocks and crystal structures of op phases for DUT-48 (k), DUT-46 (l), DUT-49 (m), DUT-50 (m) (colour codes: turquoise – copper, grey – carbon, red – oxygen, blue nitrogen; white – hydrogen; pores: orange sphere – octahedral pore, yellow sphere – tetrahedral pore, green sphere – cuboctahedral pore).

1.2. *Physical measurements*:

Adsorption isotherms of carbon dioxide in a broad range of pressures and temperatures were collected using a customized pressure adsorption system adapted to a volumetric BELSORP-HP (Microtrac MRB) device and a closed cycle helium cryostat DE-202D (ARS-Cryo). The adsorption system is designed for static physisorption experiments in the temperature range of 4 – 300 K and pressure range of 0.01 – 8000 kPa. The BELSORP-HP instrument is equipped with a turbomolecular pump and has a temperature-controlled standard volume (V_S) of 20.663 cm^3. The measurement cell was calibrated at each temperature ensuring the precise calculation of the adsorbed gas amount. The dead volume of the system was determined using helium gas with 99.999% purity. Carbon dioxide of 99.999% purity was used in all adsorption experiments. All adsorption isotherms were measured using the instrument equilibrium conditions of 0.1% of pressure change within 500 s.

1.3. *In situ PXRD in parallel to physisorption of CO_2*:

In situ PXRD studies and parallelized gas adsorption were conducted at KMC-2 beamline of the BESSY II synchrotron, operated by Helmholtz-Zentrum Berlin für Materialien und Energie.[2] A customized automated instrumentation, based on the volumetric adsorption instrument and closed-cycle helium cryostat, equipped with adsorption chamber with beryllium domes was used in all experiments.[3] PXRD patterns were measured at constant wavelength λ = 0.15406 nm (E = 8048 eV) in transmission geometry. Although the sample

holder was statically mounted in the synchrotron beam, an average crystallite size of 2 – 15 µm and use of area 2D detector (Vantec 2000, Bruker) allowed to record diffraction images with reasonable particle statistics. Each 2D image was measured with 31 s exposure. For each experiment 10 –12 mg of sample were used. In order to mask the reflections of the crystalline Be-dome, mechanical tungsten slits with 5 mm aperture were mounted on the detector cone. The obtained diffraction images were integrated using DATASQUEEZE 2.2.9[4] with further processing in FITYK 0.9 software.[5] PXRD were recorded at 195 K in the automatic mode in parallel to carbon dioxide physisorption experiment. The settings for adsorption equilibrium using equilibrium are 0.1% within 300 s. *In situ* PXRD on DUT-48 in parallel to adsorption and desorption of CO_2 at 195 K was measured using customized setup, based on laboratory powder X-ray diffractometer Empyrean-2 (PANALYTICAL GmbH), equipped with Cu-$K_{\alpha 1}$ radiation (λ = 1.54059 Å), closed-cycle helium cryostat (ARS DE-102) and volumetric adsorption instrument BELSORP-max (Microtrac MRB).

2. Results and Discussion

2.1. Analysis of in situ PXRD patterns measured in parallel to adsorption of CO_2

In situ PXRD on DUT-46 and DUT-49

Detailed analysis of the PXRD patterns of CO_2@DUT-46*cp* and CO_2@DUT-49*cp* phases, measured *in situ* shows the co-existence of *op* and *cp* phases in case of DUT-46 and very low crystallinity of the DUT-49*cp* phase. In combination with the strong broadening of the reflections, the use of Rietveld method is not feasible to refine the crystal structure of the *cp* phases in both cases. Therefore, we compared the measured PXRD pattern of CO_2@DUT-49*cp* phase with PXRD patterns calculated based on metrics of the CH_4@DUT-49*cp* phase obtained from ref [6] (Fig. S2). Surprisingly, in experimentally measured PXRD in addition to the typical most intense 111 reflection at 2θ = 4.2°, an additional reflection at 2θ = 3.56° is observed.[7] Analysis of the calculated patterns of CH_4@DUT-49*cp* phase shows no intensity at this position. However, reducing the symmetry to $P2_13$ allows for a non-zero 110 reflection at this position. The simulation of DUT-49*cp* structure in $P2_13$ space group results in 2 symmetrically independent Cu_2-paddle-wheels and two ligands. However, the simulated PXRD pattern still does not show a peak at 2θ = 3.56°. After adding of 19 CO_2 molecules per Cu paddle-wheel in case of DUT-49*cp* and 16 CO_2 molecules in case of DUT-46*cp* structures, randomly distributed in the pore system and geometrical optimization of the resulting structure in Materials Studio 5.0, the calculated PXRD patterns match well with experimentally measured (Fig. S2). This fact highlights the importance of considering the guest molecules for the structural studies of porous materials with guest-filled phases. However, the Rietveld analysis in the case of DUT-49*cp* is hampered by the low intensity-to-noise ratio and broadening of the reflections. In case of DUT-46*cp*, further analysis was hindered because of the phase mixture of *op* and *cp* phases in all PXRD patterns collected in the dataset.

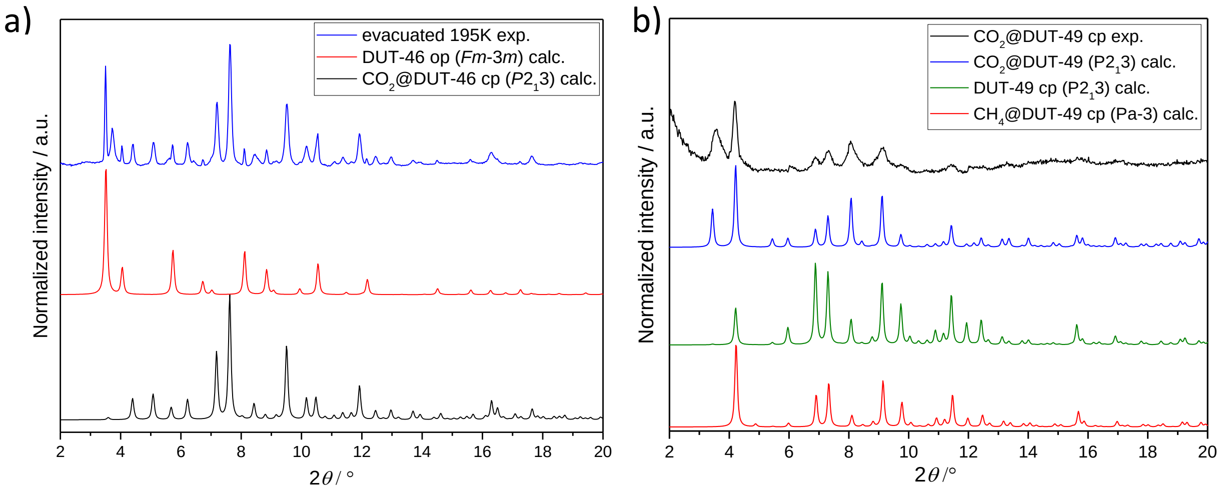

Figure S2. Simulated and experimental PXRD patterns of *cp* phases: a) DUT-46*cp*; b) DUT-49*cp*.

In situ PXRD on DUT-48

Interestingly, physisorption isotherm measured in parallel to diffraction, shows a narrow H1 hysteresis between adsorption and desorption branches in the pressure range of 0-35 kPa, which was not observed in the *ex situ* measured isotherm (Fig. S4b). The reason may be the longer equilibration time in case of *in situ* isotherm, where in addition to the equilibrium criteria, defined in the

adsorption measurement software, one hour per point was added for each measured PXRD patterns. However, the analysis of the PXRD patterns indicate no phase transitions in adsorption and desorption branch of the isotherm (Fig. S3 b,c). Variation of the reflection intensities with the CO_2 loading, clearly seen in the range of 2θ = 7-8°, indicates the ordering of the CO_2 in the pores of the framework. Comparison of the PXRD patterns, measured in adsorption and desorption branches at the similar loadings, show nearly perfect overlap of all intensities and indicate the same distribution of the CO_2 molecules in the pores (Fig. S3d-f).

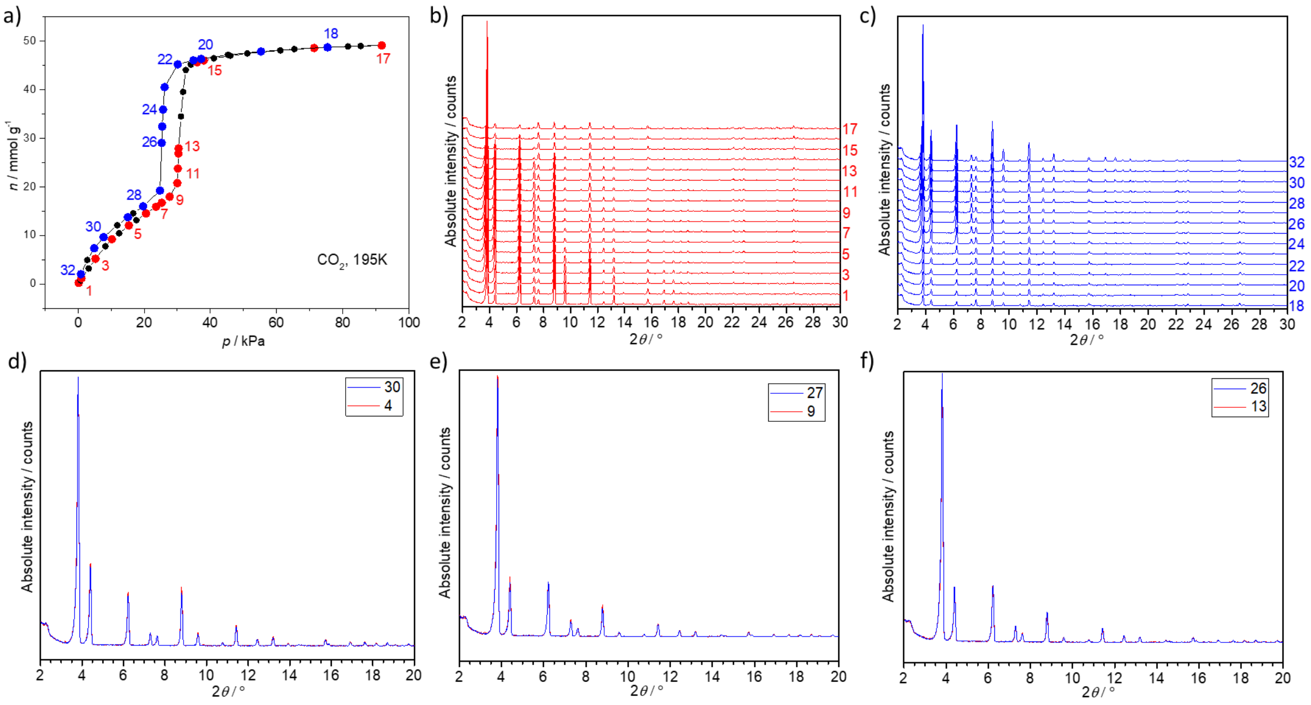

Figure S3. *In situ* PXRD on DUT-48, measured during adsorption and desorption of CO_2 at 195K: a) adsorption and desorption isotherms, measured *in situ* (red and blue points correspond to PXRD patterns measured in adsorption and desorption branch); b) PXRD patterns measured in adsorption branch; c) PXRD patterns measured in desorption branch; d-f) comparison of PXRD patterns measured during adsorption and desorption at the same CO_2 loadings.

2.2. Adsorption of CO_2 on DUT-48, DUT-46, DUT-49 and DUT-50

Pressure amplification at high absolute pressures significantly exceeding 100 kPa is observed in the CO_2 adsorption isotherms of DUT-49 (230 K and 240 K) and DUT-50 (240 K). Hence, CO_2 is a suitable fluid for proof-of-principle PA experiments. In case of DUT-49, 2.4 mmol g^{-1} CO_2 are released at 350 kPa and 230 K (Fig. S5d). Increasing the temperature to 240 K raises PA to 538 kPa, but decreases the amount of released gas to 1.7 mmol g^{-1} (Fig. S5e). The larger pores of DUT-50 shift the overloaded metastable state to even higher pressure with NGA event at 680 kPa and 240 K and Δn_{NGA} = 1.1 mmol g^{-1}, which is the highest PA experimentally ever observed (Fig. S5f). The pore volume from the CO_2 isotherms was calculated in saturation assuming that the fluid exists in the pores in the quasi-liquid state. In most of the cases, the obtained values match well with the values extracted from the nitrogen adsorption isotherms (Table S1).

Table S1. Experimental pore volumes (V_g / cm^3g^{-1}) calculated from nitrogen and carbon dioxide adsorption isotherms of DUT-48, DUT-46, DUT-49 and DUT-50 at p/p_0 = 0.8.

	Fluid density (NIST)* (cm^3g^{-1})	Pore volume DUT-48 (cm^3 g^{-1})	Pore volume DUT-46 (cm^3 g^{-1})	Pore volume DUT-49 (cm^3 g^{-1})	Pore volume DUT-50 (cm^3 g^{-1})
N_2 isotherm (77 K)	0.806	1.934	2.132	2.817	3.665
CO_2 isotherm (195 K)	1.178 (216 K)	1.906	1.183	0.762	0.819
CO_2 isotherm (220 K)	1.166	-	2.287	1.883	3.229
CO_2 isotherm (230 K)	1.128	-	2.110	2.746	2.683
CO_2 isotherm (240 K)	1.089	-	-	2.325	2.882
CO_2 isotherm (250 K)	1.046	-	-	2.369	2.997

* https://webbook.nist.gov/chemistry/fluid/

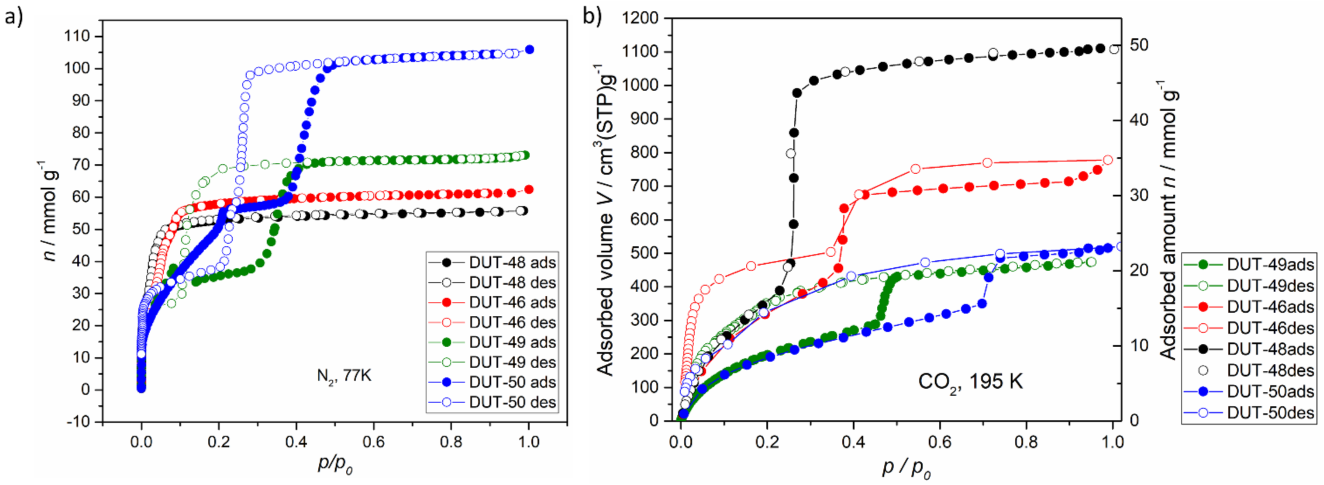

Figure S4. Adsorption and desorption isotherms of N_2 at 77 K (a) and CO_2 at 195 K (b) on DUT-48, DUT-46, DUT-49, and DUT-50.

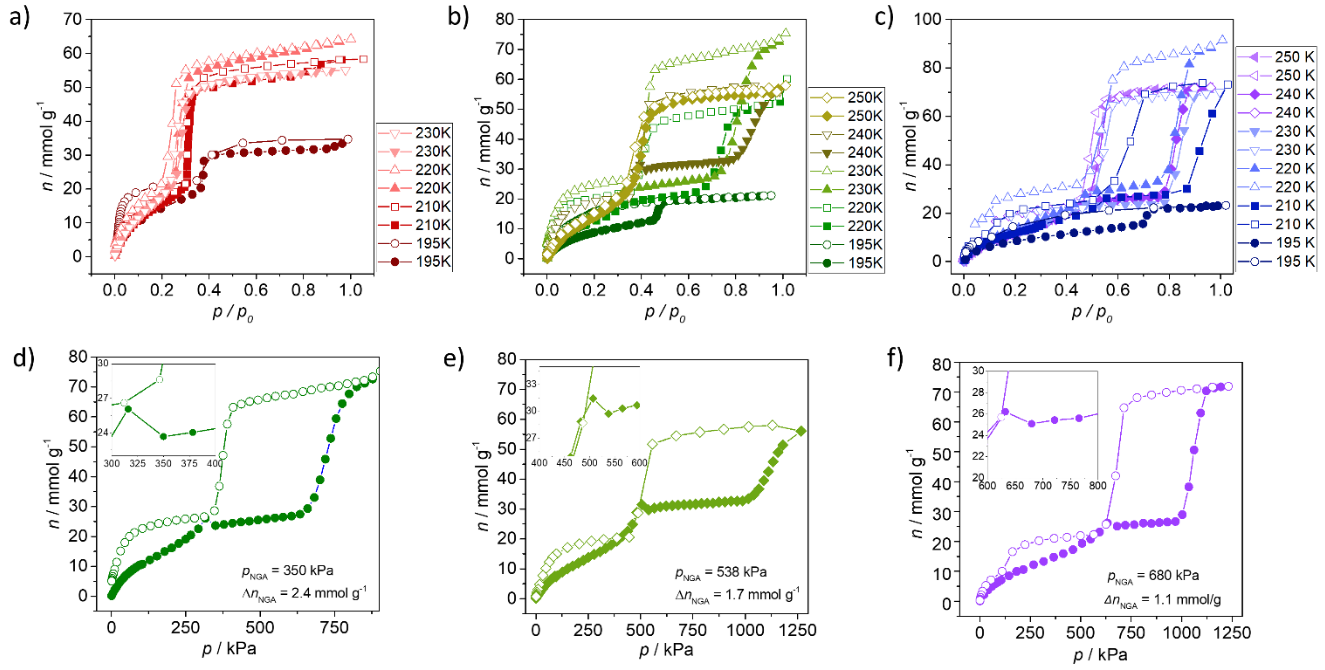

Figure S5. Adsorption and desorption isotherms of carbon dioxide on DUT-46 (a), DUT-49 (b) and DUT-50 (c) at different temperatures. Adsorption and desorption isotherms of carbon dioxide on DUT-49 at 230K (d), 240 K (e) and DUT-50 at 240 K (f).

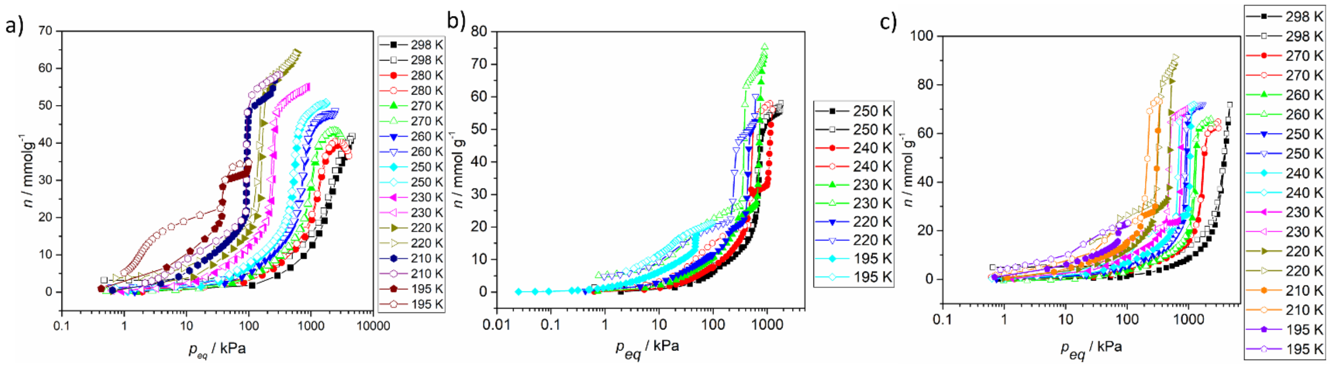

Figure S6. Semi-logarithmic plots of CO_2 adsorption and desorption isotherms on DUT-46 (a), DUT-49 (b) and DUT-50 (c) at different temperatures.

2.3. Analysis of adsorption profile for NGA

The detailed analysis of the pressure profile in the NGA range of the DUT-49 CO_2 isotherm at 230 K indicates no pressure amplification because the measurement routine of the BELSORP-HP instrument is arranged in a way that pressure is measured only with one pressure transducer, located in the standard volume section (manifold) of the instrument (Figure S7). This standard volume part (V_s = 20.663 cm^3) then sums up to the volume of the measurement cell at 230 K (V_d = 14.938 cm^3). In order to indicate the pressure amplification, a much larger amount of the sample would be needed to enhance the sample to dead volume ratio than the adsorption cell can accommodate. Thus, the sample weight of DUT-49, used in the current experiment was 0.0265 g. From the kinetic pressure profile, recorded in the experiment, the NGA step can be detected from the differences in the Δp upon adsorption. At the point before the NGA, in which DUT-49 *op* still adsorb the gas, Δp = 28 kPa (Fig. S8, S9). For the point of NGA the value decreases to 15 kPa. The next point, in which DUT-49*cp* pores are completely filled with CO_2, shows Δp = 20 kPa.

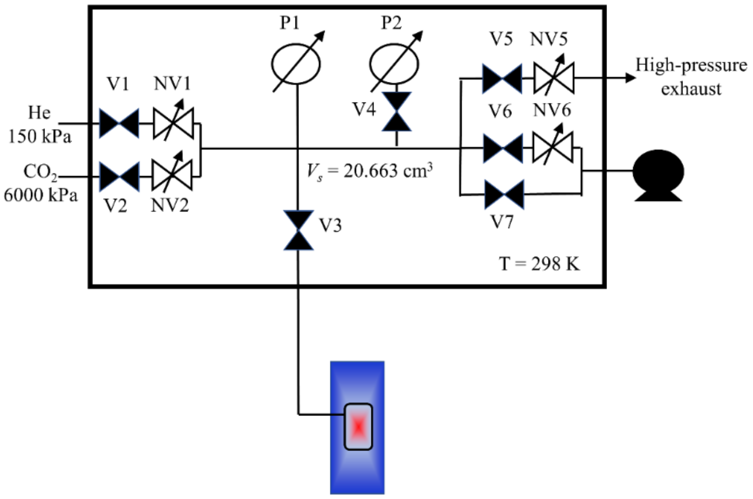

Figure S7. Schematic representation of the volumetric measurement setup.

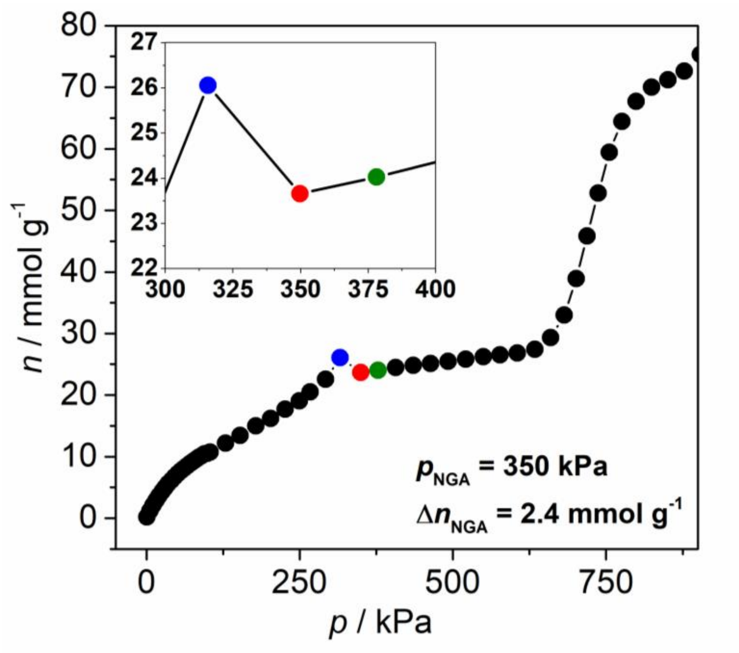

Figure S8. Adsorption of CO_2 on DUT-49 at 230 K.

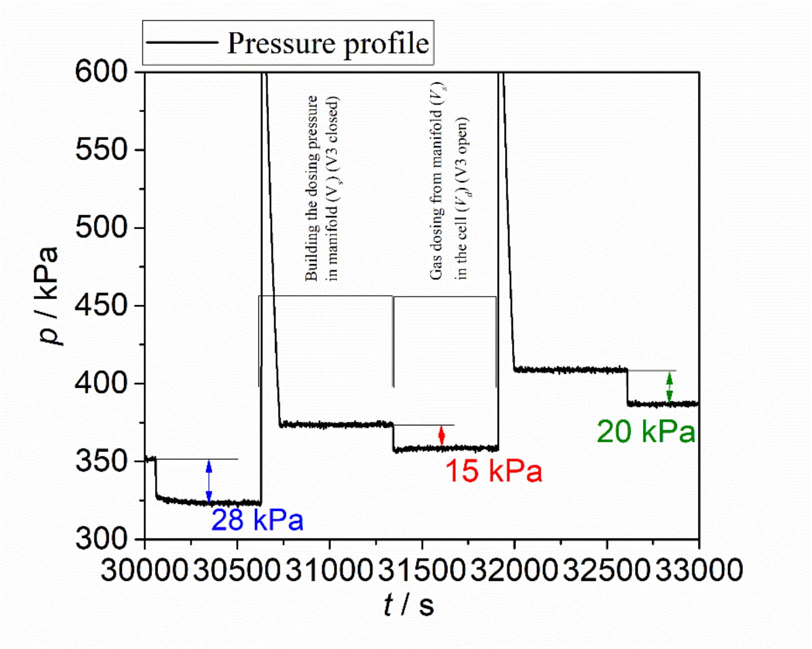

Figure S9. Pressure profile in the NGA range (colors correspond to points in Fig. S8).

2.4. Estimation of pressure amplification for DUT-49 and DUT-50.

From the adsorption isotherms and location of the PA steps we can estimate the operating window for PA materials in pressure amplification devices (Fig. S10). The lower operation point is defined by p_{NGA}, which is the absolute pressure at which NGA occurs at a given temperature. The maximum pressure amplification which can be obtained in the closed system at constant temperature is defined by p_{GO} (GO – “gate re-opening”), which is the pressure at which the framework opens the pores and consequently readsorbes gas expelled upon NGA. The maximum pressure amplification, Δp_{max} that can be achieved in a closed system at constant temperature can be estimated by the difference of p_{GO} - p_{NGA} = Δp_{max}. In an isochoric isothermal system Δp_{max} is directly proportional to the released amount of gas upon NGA, Δn_{NGA} and the increase in gas pressure, Δp can be estimated by the real gas equation (1):

$$V = Z\ \Delta n_{NGA}\ RT\ /\ \Delta p_{max} \qquad (1)$$

where V is the volume, R is universal gas constant, T - temperature (K) and Z – non-ideality correction coefficient. Based on a release of $\Delta n_{NGA}/m$(MOF) = 2.4 mmol (CO_2)/g(DUT-49) at 230 K and 350 kPa we estimate a colossal Δp_{max} = 250 kPa before reopening begins at 600 kPa if the system volume is fixed (Fig.S10a).

A comparison of Δn_{NGA} values to previously investigated gases shows that the amount of expelled carbon dioxide is smaller as compared to other fluids (Fig. S12a). However, CO_2 stands out and does not follow the linear correlation of T_{NGA} (the temperature at which Δn_{NGA} reaches a maximum) and T_c (the critical temperature of the fluid) recently established for hydrocarbons and noble gases (Fig. S12b) probably due to the high polarity of the probe molecule.[5]

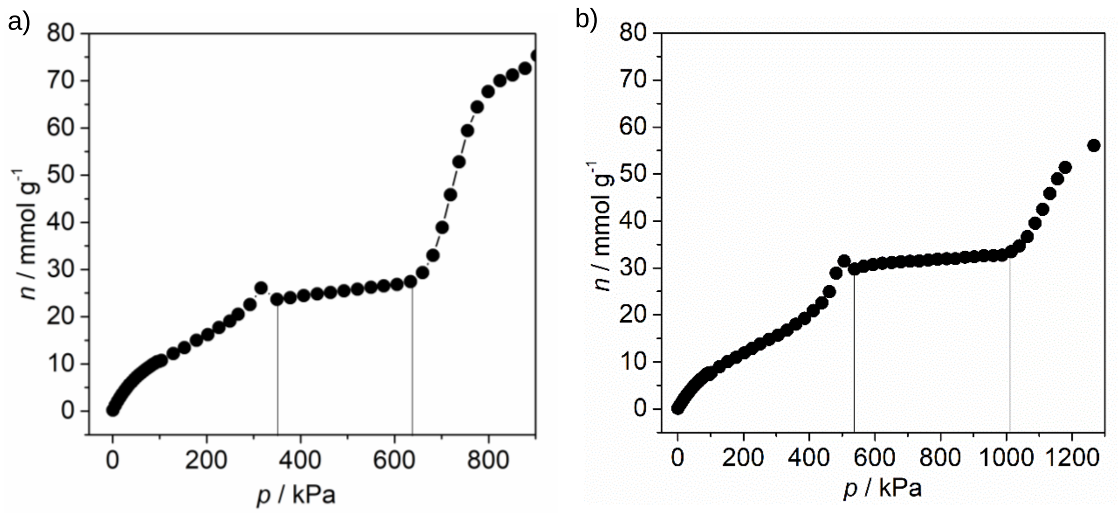

Figure S10. Adsorption isotherm of CO_2 on DUT-49 at 230 K (a) and 240 K (b).

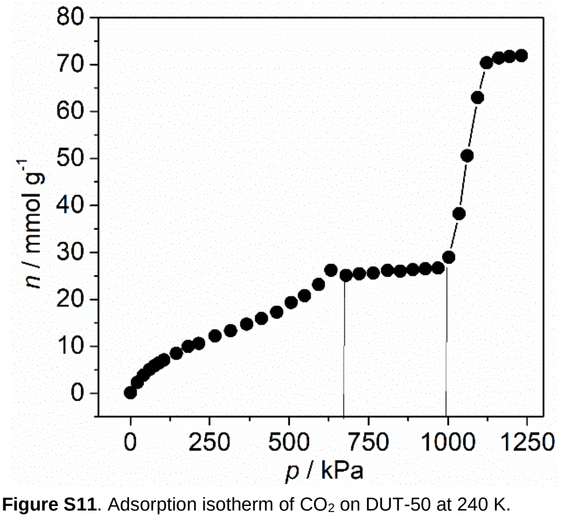

Figure S11. Adsorption isotherm of CO_2 on DUT-50 at 240 K.

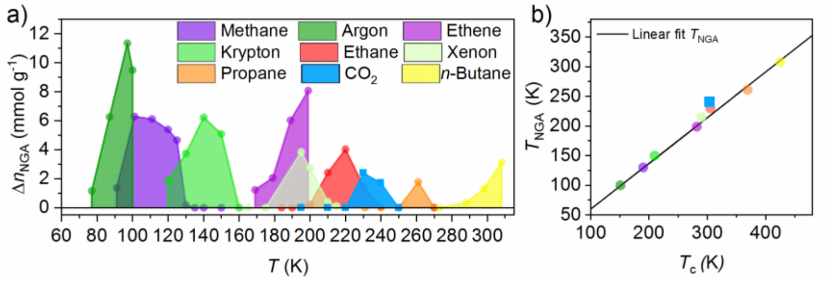

Figure S12. Correlation of Δn_{NGA} with adsorption temperature (a) and correlation of the high temperature limit at which NGA is observed with critical temperature, T_c of fluid (b) for hydrocarbons, noble gases and CO_2.

Empirical pressure amplification can be calculated using real gas equation:

pV = Z n RT, where

p – pressure in kPa, which should be achieved upon the NGA event;
V – volume of the system in L;
n – amount of gas in mol released upon NGA;
R – universal gas constant;
T – temperature in K;
Z – compressibility factor for given pressure and temperature.

The maximal gas volume of the pressure amplification can be derived as follow:

$$V = Z\ \Delta n\ RT / \Delta p$$

For the case of NGA for DUT-49 at 230 K, the pressure amplification can be derived from adsorption isotherm (Fig. S10a) as follow:

$\Delta p = p_{GO} - p_{NGA}$ = 640 kPa – 350 kPa = 290 kPa

The compressibility factor *Z* for 640 kPa of CO_2 at 230 K is 0.92.

V = 0.0024 (mol) x 8.314 (kPa L/mol K) x 230 (K) x 0.92 / 290 kPa = 0.0146 L = 14.6 cm^3

For DUT-49 at 240 K, the pressure amplification can be derived from adsorption isotherm (Fig. S10b) as follow:

$\Delta p = p_{GO} - p_{NGA}$ = 1011 kPa – 535 kPa = 476 kPa

The compressibility factor *Z* for 1011 kPa of CO_2 at 240 K is 0.89.

V = 0.0017 (mol) x 8.314 (kPa L/mol K) x 240 (K) x 0.89 / 476 kPa = 0.063 L = 6.3 cm^3
For DUT-50 at 240 K, the pressure amplification can be derived from adsorption isotherm (Fig. S11) as follow:

$\Delta p = p_{GO} - p_{NGA}$ = 986 kPa – 680 kPa = 476 kPa

The compressibility factor *Z* for 986 kPa of CO_2 at 240 K is 0.89.
V = 0.0011 (mol) x 8.314 (kPa L/mol K) x 240 (K) x 0.89 / 306 kPa = 0.064 L = 6.4 cm^3

2.5. Design of the pressure amplification experiment

In order to demonstrate the pressure amplification in the adsorption cell experimentally, we modified the measurement setup by introducing an additional electronic manometer P3 operating in the range from -100 to 2500 kPa close to the measurement cell and separated the cell and manometer from the manifold (P1 = 0 – 20 MPa, P2 = 0 - 100 kPa) by the valve V8 (Figure S13)

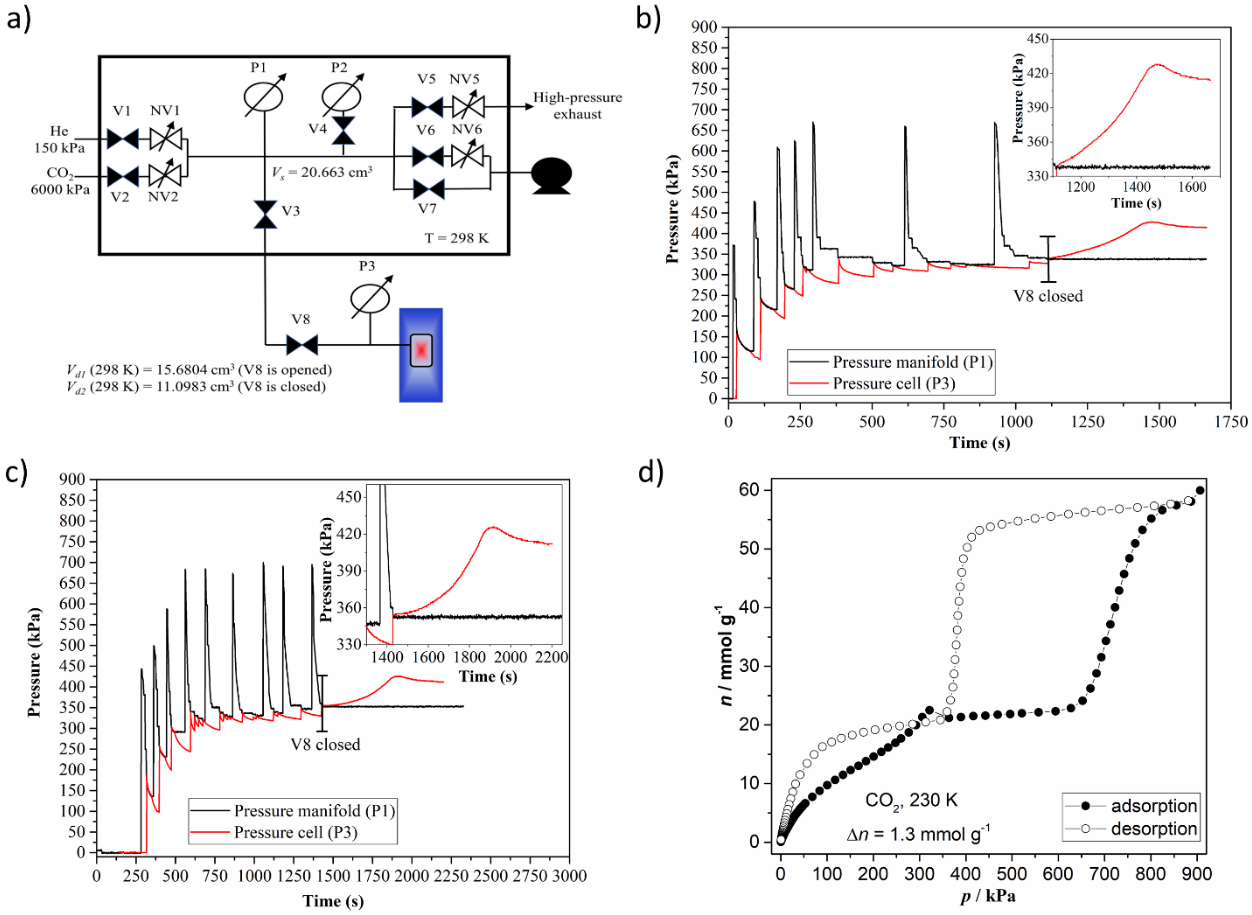

Figure S13. Schematic drawing of the custom-built amplifier (a); Pressure profiles upon NGA event in the first (b) and second (c) cycles; physisorption isotherm of CO_2 at 230 K, measured after two pressure amplification cycles (d).

As a next step the volume of the empty cell at 298 K was determined as a difference between the volumes of system with open and closed V8 valve.

$V_{298\ K}$ (V8 open) = 15.6804 cm^3
$V_{298\ K}$ (V8 closed) = 11.0983 cm^3
$V_{298\ K}$ (cell) = 4.5821 cm^3

The volume determination was performed also at the measurement temperature of 230 K with open V8:

$V_{230\ K}$ (V8 open) = 16.4676 cm^3

Later, the cell was completely filled with 165 mg of the activated DUT-49(Cu) powder, which is the maximal amount of the powder that adsorption cell can accommodate. Further the dead volume of the adsorption cell with the samples at 298 K was measured as 15.5201 cm^3.

The volume of the sample can be calculated as follows:

$V_{sample\ (298K)}$ = 15.6804 cm^3 – 15.5201 cm^3 = 0.1603 cm^3

The dead volume at 230 K can be than calculated as follows:

$V_{d\ 230\ K}$ (DUT-49(Cu)) = 16.4676 – (298K/230K) x 0.1603 cm^3 = 16.2599 cm^3

The dead volume of the measurement cell (V8 closed, sample in the cell) can be calculated as:

$V_{d\ 230K}$(cell) = $V_{d\ (230K)}$ (DUT-49(Cu)) – $V_{(298K)}$ (V8 closed) = 16.2599 cm^3 – 11.0983 cm^3 = 5.1616 cm^3 = 0.0051616 L

Since 0.165 g of DUT-49(Cu) was used in the pressure amplification experiment, the corresponding Δ*n* can be calculated (Δ*n NGA from the isotherm at 230 K is* 0.0024 mol/g):

Δ*n* = 0.0024 mol/g x 0.165 g = 0.000396 mol

From the equation
$\Delta p\ V = Z\ \Delta n\ RT$

$\Delta p = Z\ \Delta n\ RT\ /\ V$ = 0.000396 (mol) x 8.314 (kPa L/mol K) x 230 (K) x 0.92 / 0.0051616 L = 134.97 kPa

References

[1] S. Krause, J. D. Evans, V. Bon, I. Senkovska, P. Iacomi, F. Kolbe, S. Ehrling, E. Troschke, J. Getzschmann, D. M. Többens, A. Franz, D. Wallacher, P. G. Yot, G. Maurin, E. Brunner, P. L. Llewellyn, F.-X. Coudert, S. Kaskel, *Nat. Commun.* **2019**, *10*, 3632.
[2] D. M. Többens, S. Zander, *JLSRF* **2016**, *2*, A49.
[3] V. Bon, I. Senkovska, D. Wallacher, A. Heerwig, N. Klein, I. Zizak, R. Feyerherm, E. Dudzik, S. Kaskel, *Microporous Mesoporous Mater.* **2014**, *188*, 190-195.
[4] P. Heiney, Datasqueeze v.3.0.16, **2020**.
[5] M. Wojdyr, *J. Appl. Cryst.* **2010**, *43*, 1126-1128.
[6] S. Krause, V. Bon, I. Senkovska, U. Stoeck, D. Wallacher, D. M. Többens, S. Zander, R. S. Pillai, G. Maurin, F.-X. Coudert, S. Kaskel, *Nature* **2016**, *532*, 348-352.
[7] S. Krause, J. D. Evans, V. Bon, I. Senkovska, F.-X. Coudert, D. M. Többens, D. Wallacher, N. Grimm, S. Kaskel, *Faraday Discuss.* **2020**, 225, 168-183.

Author Contributions

S. Krause synthesized and characterized the MOFs. V.B. and I.S. conducted adsorption measurements and various temperatures and analyzed the data. S. Krause, V.B., D.W., N.G. and D.M.T. performed *in situ* PXRD studies under controlled gas atmosphere and analyzed the data. V.B. designed and conducted the pneumatic demonstrator experiment. S. Krause., V.B. and S. Kaskel organised and coordinated the project. All authors contributed to writing and improving the manuscript.
